# Programmed death‐ligand 1 expression and use of immune checkpoint inhibitors among patients with advanced non‐small‐cell lung cancer in a resource‐limited country

**DOI:** 10.1111/1759-7714.14442

**Published:** 2022-05-03

**Authors:** Soon Hin How, Lye Mun Tho, Chong Kin Liam, Harissa H. Hasbullah, Gwo Fuang Ho, Ibtisam Muhammad Nor, Mau Ern Poh, Kean Fatt Ho, Muthukkumaran Thiagarajan, Azlina Samsudin, Azza Omar, Choo Khoon Ong, Yong Kek Pang, Sing Yang Soon

**Affiliations:** ^1^ Kulliyyah of Medicine International Islamic University Malaysia Kuantan Malaysia; ^2^ Department of Clinical Oncology Beacon Hospital Petaling Jaya Malaysia; ^3^ Faculty of Medicine University of Malaya Kuala Lumpur Malaysia; ^4^ Oncology Unit, Faculty of Medicine Universiti Teknologi Mara Sungai Buloh Malaysia; ^5^ Oncology and Radiotherapy Department General Hospital Kuala Lumpur Kuala Lumpur Malaysia; ^6^ Mount Miriam Cancer Hospital Penang Malaysia; ^7^ Hospital Sultanah Nur Zahirah Kuala Terengganu Malaysia; ^8^ Respiratory Unit, Medical Department Hospital Raja Perempuan Zainab II Kota Bharu Malaysia; ^9^ Gleneagles Penang George Town Malaysia; ^10^ Sarawak Heart Centre Kota Samarahan Malaysia

**Keywords:** PDL‐1, immunotherapy, lung cancer

## Abstract

**Introduction:**

Immune checkpoint inhibitor (ICI) therapy is an established treatment for advanced non‐small‐cell lung cancer (NSCLC) and programmed death ligand‐1 (PD‐L1) expression is a recognized biomarker to determine response to therapy. We retrospectively analyzed NSCLC patients in the Malaysia Lung Cancer Registry (MLCR) and report on the clinical characteristics associated with PD‐L1 expression and ICI use in Malaysia, a low‐ to middle‐income country.

**Methods:**

All 901 NSCLC patients in the MLCR who were diagnosed from January 1, 2017 to December 31, 2020 from 14 hospitals across the country were analyzed.

**Results:**

Out of 901 patients, 505 had PDL‐1 testing done with complete data available only in 489 patients. The most common histology was adenocarcinoma (84.7%) followed by squamous cell carcinoma (10.2%). The majority (95%) presented with stage 3 or 4. The number and percentage of patients with PDL‐1 tumor proportion scores of ≥50%, 1–49%, and <1% were 138 (28.2%), 158 (32.3%), and 193 (39.5%), respectively. In multivariate analysis, the presence of genomic mutation is the only independent characteristic associated with negative PD‐L1 expression (crude odds ratio 0.579, 95% confidence interval 0.399–0.840, *p* = 0.004). Of 292 patients eligible for ICI therapy, only 100 patients (34.2%) received ICIs. Seventy‐eight patients received ICI therapy as first‐line treatment, 15 patients as second‐line treatment, and 7 patients as third‐line treatment.

**Conclusions:**

This is the first analysis on PD‐L1 expression and ICI use in Malaysia. Despite the proven efficacy of ICI therapy, only 56% of our patients had PD‐L1 tests performed and only 34.2% of eligible patients received ICIs.

## Graphical abstract

We retrospectively analyzed 901 non‐small‐cell lung cancer patients in the Malaysia Lung Cancer Registry and report on the clinical characteristics associated with programmed death ligand‐1 (PD‐L1) expression and immune checkpoint inhibitor use in Malaysia, a low to middle income country. The majority (95%) of our patients presented with stage 3 or 4. Only 505 had PDL‐1 testing done, with complete data available in 489 patients. The number and percentage of patients with PDL‐1 tumor proportion scores of ≥50%, 1–49%, and <1% were 138 (28.2%), 158 (32.3%), and 193 (39.5%), respectively. In multivariate analysis, the presence of genomic mutation is the only independent characteristic associated with negative PD‐L1 expression (crude odds ratio 0.579, 95% confidence interval 0.399–0.840, *p* = 0.004). Of 292 patients eligible for immune checkpoint inhibitor (ICI) therapy, only 100 patients (34.2%) received ICIs. We propose strategies to improve uptake of ICI therapy in countries with limited resources.

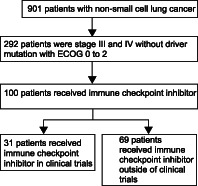

## INTRODUCTION

Lung cancer is one of the most common cancers in Malaysia, accounting for about 10% of all cancers.^1^ The majority of lung cancer patients in Malaysia are diagnosed in late stages of the disease, with 90% having stages 3 or 4 at presentation. Lung cancer is the most common cause of cancer‐related death in Malaysia, accounting for almost 20% of all cancer deaths.

Treatment of advanced stage non‐small‐cell lung cancer (NSCLC) without driver mutation centers around immune check point inhibitor (ICI) therapy with or without conventional chemotherapy.^2^ Single‐agent ICIs have been shown to improve outcomes over conventional chemotherapy in NSCLC patients with high programmed death‐ligand 1 (PD‐L1) expression.^3^ The Keynote‐024 study showed that first‐line pembrolizumab monotherapy in patients with PD‐L1 expression >50% resulted in an overall survival (OS) of 30 months compared to 14.2 months with first‐line chemotherapy alone.^4^ Similarly, in the IM power 110 study, first‐line atezolizumab monotherapy is associated with better outcomes compared to chemotherapy in advanced NSCLC patients with high PD‐L1 expression.^5^ Combination of ICIs with chemotherapy improves outcomes even further, extending benefit to those with low or no PD‐L1 expression. In Keynote‐189, first‐line pembrolizumab with chemotherapy improves the median OS compared to chemotherapy alone regardless of PD‐L1 expression.^6^ In IMpower 150, atezolizumab in combination with chemotherapy and bevacizumab significantly improved outcomes as first‐line treatment in nonsquamous metastatic NSCLC.^7^

Although not as perfect as driver mutation as a predictive biomarker, PD‐L1 is used as a biomarker to predict response to treatment with ICIs.^2^ In Malaysia, PD‐L1 expression using immunohistochemistry (IHC) has been performed since 2016. A national consensus guideline on molecular testing published in 2019 recommends PD‐L1 testing for NSCLC patients with no druggable driver mutations.^8^ In this study, we interrogate the lung cancer database in the Malaysian Lung Cancer Registry (MLCR) to determine the prevalence of PD‐L1 expression in our NSCLC patients and their demographic and clinical characteristics associated with PD‐L1 expression.

There are challenges in making ICI therapy universally available in resource‐limited settings. Malaysia, being a low‐ to middle‐income country, faces such issues and the secondary aim of this study was to document the usage and accessibility of ICI therapy in our NSCLC patients registered in the MLCR.

## METHODS

The MLCR was set up in 2015 through a collaborative effort of 14 major hospitals including public, university, and private tertiary referral centers across Malaysia. Investigators from these participating hospitals enter data in the online registry using an electronic case report form (eCRF) designed to capture demographic, clinical, treatment, and outcome data. Data collected includes patients′ baseline characteristics (age, sex, ethnicity, smoking status, Eastern Cooperative Oncology Group [ECOG] performance status and comorbid illnesses), staging, NSCLC histology, molecular test results, treatment, and survival outcome. Patients with driver mutations were those with epidermal growth factor receptor (*EGFR*) mutation, anaplastic lymphoma kinase (*ALK*) rearrangement, or ROS1 rearrangement. Clinical staging was based on the eighth edition of TNM classification.^9^ The histology of NSCLC was divided into adenocarcinoma, squamous cell carcinoma (SCC), and others, including adenosquamous carcinoma, large cell carcinoma, and NSCLC‐not otherwise specified (NSCLC‐NOS).

We retrospectively analyzed the PD‐L1 status of patients with NSCLC diagnosed during the period from January 1, 2017 to December 31, 2020. In Malaysia, only two antibodies are used for determining the PD‐L1 expression of NSCLC, namely, 22C3 using the Dako platform and SP263 using the Ventana platform. All molecular and PD‐L1 testing was conducted in accredited laboratories with appropriately trained pathologists. The 22C3 and SP263 antibodies have been shown to have similar performance in determining PD‐L1 expression of tumor cells.^10^, ^11^ In this study, PD‐L1 expression levels were considered the same irrespective of the platform and antibody used. All PD‐L1 levels reported in this study refer to the tumor proportion score (TPS). Positive PD‐L1 expression is when the TPS is ≥1%, which is further subdivided into 1–49% and ≥50%.

When analyzing patients treated with ICI therapy, only patients with either stage 3 or 4 NSCLC without driver mutation and ECOG performance status 0–2 were included. Patients enrolled in clinical trials were included in the analysis as well.

This study was approved by the University of Malaya Medical Research Ethics Committee. Individual patient informed consent was considered unnecessary as the information in the MLCR database was treated with confidentiality and accessed using patient identification codes which were completely anonymized.

### Statistical analysis

Data were collated and extracted from the MLCR in Microsoft Excel 2019 format. Data files were transformed into comma separated value (.csv) file before exporting into SPSS (.spv) files to avoid data transportation errors. Any missing data entry was identified and the site investigator contacted to resolve the issue. Statistical analysis was performed using IBM SPSS Statistics version 25.0. In descriptive statistics, frequency and valid percentage were used to describe categorical data. Mean and standard deviation were used to describe normally distributed continuous variables.

Univariate analysis was performed using Pearson chi‐square and simple logistic regression to obtain crude odds ratios (ORs), 95% confidence intervals (CIs), and *p* values. Independent variables found to be significant in univariate analyses and deemed clinically important were subjected to multivariate analysis using multiple logistic regression. The significance level was set at *α* < .05 for two‐sided tests.

## RESULTS

For the study period from January 1, 2017 to December 31, 2020, 901 patients with NSCLC were registered in the registry. Table 1 summarizes the demographic and clinicopathological characteristics of all the patients diagnosed with NSCLC during this study period. The most common histology was adenocarcinoma (86.2%) followed by squamous cell carcinoma (9.4%). The majority (85.4%) were diagnosed with stage 4 disease followed by stage 3 disease (9.4%). Only 5.2% were diagnosed with stage 1 or 2 disease. At diagnosis, the majority of the patients had ECOG performance status of 1 (51.2%) and only 14.7% had ECOG performance status of 3 or 4.

**TABLE 1:** Demographic and clinicopathological characteristics of all patients, *n* = 901

Variable	Total
	*n* (%)
Sex	
Male	482 (53.5)
Female	419 (46.5)
Smoking history^a^ (*n* = 892)	
Never smoker	565 (63.3)
Smoker (former and current)	327 (36.7)
Ethnicity	
Malay	435 (48.2)
Chinese	355 (39.4)
Others	111 (12.3)
Histology	
Adenocarcinoma	776 (86.2)
Squamous cell carcinoma	85 (9.4)
Adenosquamous	22 (2.4)
Others	18 (2.0)
Stage^a^ (*n* = 815)	
1	26 (3.2)
2	16 (2.0)
3	77 (9.4)
4	696 (85.4)
ECOG performance status^a^ (*n* = 892)	
0	185 (20.7)
1	456 (51.2)
2	120 (13.4)
3	88 (9.9)
4	43 (4.8)
Biomarker tested	
*EGFR*	826 (91.7)
*ALK*	454 (50.4)
*ROS1*	268 (29.7)
*BRAF*	25 (2.8)
*MET* (including *MET* amplification and *MET* exon 14 skipping	19 (2.1)
*HER2*	14 (1.6)
*KRAS*	24 (2.7)
PD‐L1	505 (56.0)

a *n* not 901 due to missing data.

*EGFR* mutation (826 patients, 91.7%) was the most common biomarker tested, with half of the patients (50.4%) testing positive for this mutation. The second most common genomic test performed was *ALK* rearrangement, which yielded positive results in 6.1% of 454 patients tested. *ROS‐1* rearrangement was tested in 268 patients (29.7%) and was detected in only 1.1% of them. Other rare driver mutations tested were *BRAF* (2.8%), *KRAS* (2.7%), *MET exon 14 skipping* (2.1%), and *HER2* (1.6%). Molecular biomarker testing was not performed in 65 patients (0.9%). PD‐L1 expression was determined in 505 patients (56.0%).

### PD‐L1 expression

**FIGURE 1:**
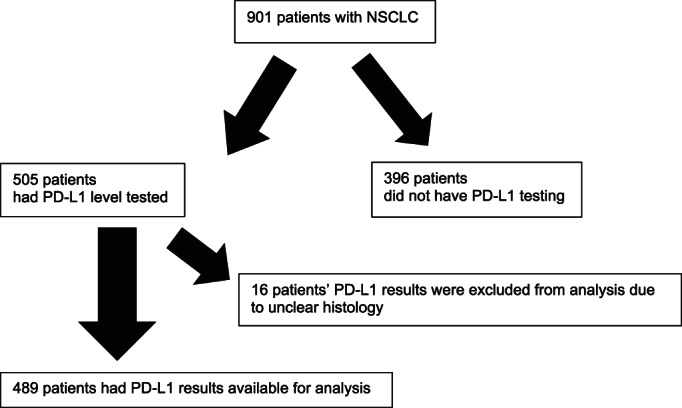
Programmed death ligand‐1 (PDL‐1) testing in non‐small‐cell lung cancer (NSCLC) patients

Among 505 patients with PD‐L1 expression determined, 16 patients had unclear histology and were excluded from further analysis (Figure 1), therefore only 489 patients were further analyzed. Of the two antibodies used to determine PD‐L1 expression, 22C3 was the most frequently used (74.5%) followed by SP263 (25.5%). Table 2 shows the demographic and clinical characteristics of the 489 patients with PD‐L1 results. The mean age (+SD) of the patients was 63.5 ± 10.8 years. Slightly more than half were male. There were about equal numbers of Malay and Chinese patients.

**TABLE 2:** Characteristics of patients with positive or negative PD‐L1 expression, *n* = 489

Variable	Positive PD‐L1	Negative PD‐L1	*χ* ^2^ (df)	*p* value
	*n* (%)	*n* (%)		
Sex				
Male	179 (62.4)	108 (37.6)	0.982 (1)	0.322
Female	117 (57.9)	85 (42.1)		
Smoking history^a^ (*n* = 482)				
Never smoker	160 (57.6)	118 (42.4)	2.183 (1)	0.140
Smoker (former and current)	131 (64.2)	73 (35.8)		
Ethnicity				
Malay	133 (59.6)	90 (40.4)	1.639 (2)	0.441
Chinese	146 (62.7)	87 (37.3)		
Others	17 (51.5)	16 (48.5)		
Histology				
Adenocarcinoma	240 (58.0)	174 (42.0)	7.436 (2)	**0.024**
Squamous cell carcinoma	37 (74.0)	13 (26.0)		
Others	19 (76.0)	6 (24.0)		
Stage^a^ (*n* = 434)				
1 or 2	11 (50.5)	11 (50.0)	1.090 (2)	0.296
3 or 4	252 (61.2)	160 (38.8)		
Genomic mutation^a^ (*n* = 479)				
Negative	183 (65.8)	95 (34.2)	8.354 (1)	**0.004**
Positive	106 (52.7)	95 (47.3)		
ECOG performance status^a^ (*n* = 482)				
0–1	218 (61.2)	138 (38.8)	0.423 (2)	0.515
2–4	73 (57.9)	53 (42.1)		

*Notes*: Positive PD‐L1 expression = tumor proportion score (TPS) >1%, negative PD‐L1 expression = TPS <1%. Significant *p* values in bold.

a Evaluated for patients with confirmed cases of smoking history, final staging, genomic mutation, and ECOG status.

Of the 489 patients, 193 (39.5%) had tumors with no PD‐L1 expression. The TPS was >50% in 138 (28.2%) patients and 1–49% in the remaining 158 (32.3%) patients. There was no statistical difference in PD‐L1 expression between the different ethnic groups (Tables 2 and 3).

**TABLE 3:** Univariate analysis: Characteristics of patients according to PD‐L1 expression, *n* = 489

Variable	PD‐L1 tumor proportion score			*χ* ^2^ (df)	*p* value
	<1%	1–49%	≥50%		
Sex					
Male	108 (37.6)	93 (32.4)	86 (30.0)	1.345 (2)	0.510
Female	85 (42.1)	65 (32.2)	52 (25.7)		
Smoking history* (*n* = 482)					
Nonsmoker	118 (42.4)	92 (33.1)	68 (24.5)	4.377 (2)	0.112
Smoker (ex and current)	73 (35.8)	64 (31.4)	67 (32.8)		
Ethnicity					
Malay	90 (40.4)	68 (30.5)	65 (29.1)	2.175 (4)	0.704
Chinese	87 (37.3)	81 (34.8)	65 (27.9)		
Others	16 (48.5)	9 (27.3)	8 (24.2)		
Histology					
Adenocarcinoma	174 (42.0)	126 (30.4)	114 (27.5)	9.751 (4)	**0.045**
Squamous cell carcinoma	13 (26.0)	19 (38.0)	18 (36.0)		
Others	6 (24.0)	13 (52.0)	6 (24.0)		
Stage* (*n* = 434)					
1–2	11 (50.0)	9 (40.9)	2 (9.1)	4.149 (2)	0.126
3–4	160 (38.8)	132 (32.0)	120 (29.1)		
Genomic mutation* (*n* = 479)					
Negative	95 (34.2)	92 (33.1)	91 (32.7)	10.090 (2)	**0.006**
Positive	95 (47.3)	62 (30.8)	44 (21.9)		
ECOG* (*n* = 482)					
0–1	138 (38.8)	115 (32.3)	103 (28.9)	0.637 (2)	0.714
2–4	53 (42.1)	41 (32.5)	32 (25.4)		

*Note*: Significant *p*‐value in bold. *Evaluated for patients with confirmed smoking history, final staging, genomic mutation and ECOG performance status.

On univariate analysis, the tumors of patients with adenocarcinoma or driver mutation‐positive were more likely to have no PD‐L1 expression compared to those with non‐adenocarcinoma histology or driver mutation‐negative (Tables 2 and 3). However, on multivariate analysis, the presence of driver mutation was the only independent characteristic associated with the absence of PD‐L1 expression (crude OR 0.579, 95% CI 0.399–0.840, *p* = 0.004 (Table 4).

**TABLE 4:** Multivariate analysis of clinical characteristics associated with positive or negative PD‐L1 expression using logistic regression, *n* = 489

Variable	Simple logistic regression	
	Crude OR (95% CI)	*p* value
Sex		
Female (reference)	1	–
Male	1.204 (0.834, 1.739)	0.322
Smoking history		
Nonsmoker (reference)	1	–
Smoker (ex and current)	1.323 (0.912, 1.920)	0.172
Ethnicity		
Others (reference)	1	–
Malay	1.391 (0.668, 2.895)	0.378
Chinese	1.579 (0.759, 3.286)	0.221
Histology		
Others (reference)	1	–
Adenocarcinoma	0.436 (0.170, 1.113)	0.083
Squamous cell carcinoma	0.899 (0.295, 2.739)	0.851
Stage		
1–2 (reference)	1	–
3–4	1.575 (0.667, 3.718)	0.300
Genomic mutation		
Negative (reference)	1	–
Positive	0.579 (0.399, 0.840)	**0.004**
ECOG performance status		
0–1 (reference)	1	–
2–4	0.872 (0.577, 1.318)	0.515

*Note*: Significant *p*‐value in bold.

### Use of ICI therapy

**FIGURE 2:**
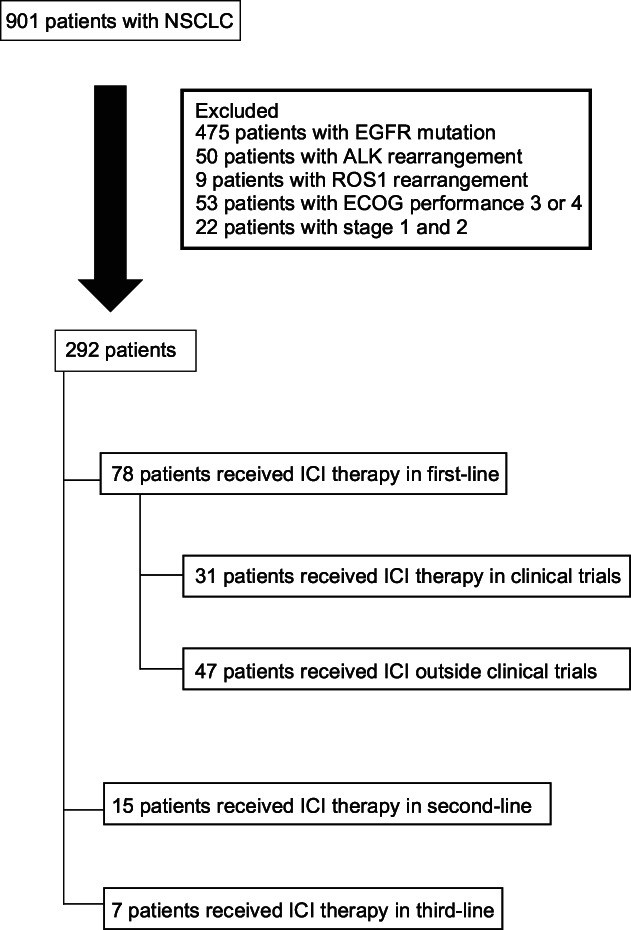
Analysis of non‐small‐cell lung cancer (NSCLC) patients who are ECOG 0–2 and without driver mutation who received immune checkpoint inhibitors (ICIs)

Of the 901 patients with NSCLC diagnosed during the study period, we examined the use of ICI therapy in 292 patients with either stage 3 or stage 4 disease without driver mutation and with good ECOG performance status of 0–2 (Figure 2). Of these 292 patients, only 100 (34.2%) received ICI therapy.

From these 100 patients who received ICI, 31 patients received ICI in multicenter clinical trials as first‐line treatment (Figure 2). Of the remaining 69 patients who received ICI therapy outside of clinical trials, the majority (47 patients) also received ICIs as first‐line therapy with the majority (31 patients) receiving ICIs in combination with chemotherapy. Fifteen patients received ICI therapy as second‐line treatment and the remaining seven patients received ICI therapy as third‐line treatment. No patient received ICI therapy as fourth‐line treatment or beyond.

Pembrolizumab was the most commonly used ICI in the first‐line setting (38 of 47 patients, 80.1%). No patient was given 35 doses of pembrolizumab despite some patients showing good response to pembrolizumab. Atezolizumab and durvalumab were used much less frequently. Almost all patients treated with ICI had PD‐L1 tested, except two patients: one received durvalumab consolidation after chemoradiotherapy for stage 3 disease and another patient received a combination of ICI and chemotherapy.

We did not collect data on household income, but we noted that of those 69 patients who received ICI outside of clinical trials, the majority were male (49 patients (71%)) Their ages ranged between 39 and 87 years old, with mean age of 67 years. Chinese and Malay patients were almost equally represented (49.3% and 44.9%), respectively.

## DISCUSSION

PD‐L1 expression by IHC has been extensively evaluated in clinical trials as a predictive biomarker for patients with advanced NSCLC. In Malaysia, only two antibodies were used to determine PD‐L1 TPS, 22C3 and SP263. The analytic performance and concordance of the different assays were evaluated previously and showed the 22C3 and SP263 PD‐L1 assays are highly concordant, whereas the SP142 assay is less sensitive for staining of both tumor cells and tumor‐infiltrating immune cells.^10^, ^11^ We therefore combined the PD‐L1 results of patients determined by both the 22C3 and SP263 assays in our analysis.

In our study patients, the prevalences of PD‐L1 TPS ≥50%, 1–49%, and <1% were 28.2%, 32.3% and 39.5%, respectively which is consistent with the findings of the global multicenter EXPRESS study with corresponding prevalences of 22%, 30%, and 48%, respectively.^12^ In the EXPRESS study, there is no difference in PD‐L1 expression between tumors with driver mutations and those without. Our study, however, shows that tumors with driver mutation are more likely to have no PD‐L1 expression.

In another study of lung cancer in an East Asian population, Pan et al.^13^ showed that high PD‐L1 TPS ≥50% is significantly associated with male gender, current/ex‐smoker, and SCC histology. However, in our multivariate analysis, only patients without driver mutation are likely to have PD‐L1 expression. Pan et al. also concluded that the extremely low prevalence of PD‐L1 expression (4.1%) among their adenocarcinoma patients could be attributed to a higher proportion of never smokers (62%) in their cohort.^13^ However, while the percentage of our patients who were never smokers and the percentage of adenocarcinoma in our patients were high at 57.7% and 84.7%, respectively, PD‐L1 expression was noted in about 57.6% of our patients who were never smokers and 58% of those with adenocarcinoma.

In Malaysia, the combination of ICI and chemotherapy is registered as a first‐line treatment in advanced NSCLC regardless of PD‐L1 expression, whereas pembrolizumab monotherapy is a first‐line treatment option in patients with PD‐L1 expression >1%. Atezolizumab, nivolumab, and pembrolizumab are available as second‐line treatments in patients who failed previous chemotherapy. Durvalumab can be given after chemoradiotherapy in stage 3 NSCLC. However, only a small number of our patients received ICI as part of their treatment despite having criteria to receive ICI. We hypothesized that cost is the main deterrent. We did not specifically collect data on household income. However, according to the 2019 socioeconomic statistics report, the median monthly household income in Malaysia was USD1400,^14^ which is too little to bear the cost of ICI therapy, which ranges from US$2400 to US$4200 every 3 weeks excluding the costs of medical consultation and hospitalization. Currently, the treatment is not subsidized or reimbursable by the government. Many of our patients received ICI therapy as part of a clinical trial (31%). Others are self‐paying or claiming from private insurance.

As expected, patients who had PD‐L1 tested were more likely to receive immunotherapy compared to patients without PD‐L1 tested. This is likely because as most of the patients could not afford ICI therapy, the treating physicians did not order PD‐L1 testing for them. Almost half of our patients who were treated with ICIs received the treatment in clinical trials. Otherwise, the number of patients treated with ICIs would have been much fewer. Another reason for not determining PD‐L1 expression was because ICI therapy can be combined with chemotherapy in the first‐line treatment of advanced NSCLC regardless of the PD‐L1 status.

Our data confirm our clinical suspicion and observation that only a limited number of patients across different hospital settings in our country receive ICI therapy despite overwhelming evidence of its benefit in metastatic NSCLC patients.

Apart from entry into clinical trials, we propose two further possible approaches to improve uptake of ICI therapy in our metastatic NSCLC patients. First, it is perhaps possible to use shorter course of treatment instead of the long 35 courses. Sukari and Nagasaka reported a patient who had only two cycles of pembrolizumab plus chemotherapy but further shrinkage of the primary tumor was observed 2 years later with no evidence of extrathoracic metastasis.^15^ Yilmaz and Guven et al. reported two cases of durable response after 4 and 9 months of nivolumab, respectively, and both cases did not suffer a relapse for 4 years after diagnosis^16^ (Yilmaz, pers. comm., 2021). These case series suggest that limited doses of ICI therapy may benefit patients who cannot afford the recommended 35 doses.

Second, it is perhaps possible to use lower the dose of ICI in patients who have financial constraints. Atezolizumab has shown clinical activity doses ranging from 1 to 20 mg/kg in phase 1 studies while 15 mg/kg was used in a phase 3 study.^17^ The Keynote 001 study also showed pembrolizumab to have similar efficacy at 2 mg/kg 3‐weekly and 10 mg/kg 3‐weekly.^18^ Further studies in developing countries are needed to look at the efficacy and safety of lower doses or less frequent dosing of ICIs in advanced NSCLC patients.

Our data will help us to secure further funding to study these two proposed alternative approaches in our patient population.

About 16% of our patients who received first‐line ICI therapy with or without chemotherapy had an ECOG performance status of 2. It is not uncommon in the real world for patients with ECOG performance status of 2 to be treated with ICI therapy, although such patients are excluded from randomized clinical trials. The CheckMate 171 study is one of the clinical trials which enrolled SCC patients with ECOG performance status of 2 to receive treatment with nivolumab after failure of first‐line systemic therapy. The results confirmed patients with ECOG 2 also seem to benefit from ICI therapy.^19^ Friedlaender et al. retrospectively analyzed the outcomes of advanced NSCLC patients with high PD‐L1 expression (>50%) treated with first‐line pembrolizumab, comparing patients with ECOG performance status 2 versus those with ECOG performance status 0–1, and showed that the median OS and progression‐free survival (PFS) were shorter in patients with ECOG performance status 2 but there was no significant difference in treatment‐related toxicity between the two groups.^20^ Ahmed et al., in another retrospective study of 285 NSCLC patients treated with various ICIs, also showed that the median OS and PFS of patients with ECOG performance status 2 (5.1 and 8.3 months, respectively) were shorter than in patients with ECOG performance status 0 or 1 (7.4 and 14.7 months, respectively).^21^ However, the authors concluded that ECOG performance status 2 did not constitute grounds for disqualifying these patients from ICI therapy. Alessi et al. observed a response rate of 25.6% in ECOG performance status 2 patients, which is comparable with the reported response rate of platinum doublet chemotherapy.^19^ Pembrolizumab monotherapy generally has a more favorable side‐effect profile and potential durable response, suggesting that ICI therapy may be a better option in patients with ECOG performance status 2 and high PD‐L1 expression.

## CONCLUSIONS

In conclusion, half of our patients with NSCLC had PD‐L1 expression determined and the prevalences of PD‐L1 TPS ≥50%, 1–49%, and <1% were 28.2%, 32.3%, and 39.5%, respectively. Despite data showing the efficacy of ICI therapy, only 34.2% of advanced NSCLC patients without driver mutation and with ECOG performance status 0–2 received ICI therapy because of financial constraints. Patients with PD‐L1 expression determined were more likely to be treated with ICIs.

## CONFLICT OF INTEREST

All authors declare no conflict of interest pertaining to this manuscript

## DISCLOSURE

All authors declare no conflict of interest pertaining to this manuscript.
